# Clinical and microbiological characteristics of nosocomial, healthcare-associated, and community-acquired *Klebsiella pneumoniae* infections in Guangzhou, China

**DOI:** 10.1186/s13756-021-00910-1

**Published:** 2021-02-25

**Authors:** Tingting Le, Ling Wang, Chaoying Zeng, Leiwen Fu, Zhihua Liu, Jing Hu

**Affiliations:** 1 Department of Nosocomial Infection Administration, Zhujiang Hospital, Southern Medical University, Guangzhou, 510282 Guangdong China; 2 Department of Laboratory Medicine, Zhujiang Hospital, Southern Medical University, Guangzhou, 510282 Guangdong China; 3 School of Public Health (Shenzhen), Sun Yat-Sen University, Shenzhen, 518000 Guangdong China; 4 Department of Infectious Disease, Nanfang Hospital, Guangzhou, 510282 Guangdong China

**Keywords:** *Klebsiella pneumoniae* infections, Nosocomial, Healthcare-associated, Community-acquired, Antimicrobial resistance

## Abstract

**Background:**

*Klebsiella pneumoniae* (*K. pneumoniae*) is a common pathogen associated with hospital and community-onset infections. This study aimed to compare the clinical and microbiological characteristics of nosocomial, healthcare-associated (HCA), and community-acquired (CA) *K. pneumoniae* infections.

**Methods:**

Clinical data were extracted from electronic medical records and analyzed retrospectively. Antimicrobial susceptibility and extended-spectrum beta-lactamase (ESBL) production were determined for all identified strains. Carbapenemase and ESBL genes were amplified by PCR. Genotyping of carbapenem-resistant *K. pneumoniae* (CRKP) and ESBL-producing strains was performed by pulsed-field gel electrophoresis (PFGE).

**Results:**

Of 379 *K. pneumoniae* infections, 98 (25.9%) were nosocomial, 195 (51.5%) were healthcare-associated, and 86 (22.6%) were community-acquired. Hematological malignancy (OR = 4.467), and hypertension (OR = 2.08) and cerebral vascular disease (OR = 2.486) were associated with nosocomial and HCA infections respectively, when compared to CA infections. Overall, the incidence of antimicrobial resistance for the majority of agents tested was similar between nosocomial and HCA infections (*P* > 0.05) and both groups had a higher incidence than CA infections (*P* < 0.05). Moreover, 95.1% (78/82) of CRKP strains were isolated from the nosocomial and HCA groups. The *bla*_KPC_ was the most prevalent carbapenemase gene among CRKP strains (80.5%, 66/82). ESBL-producing strains were prevalent among nosocomial (40.8%), HCA (35.9%) and CA groups (24.4%). The *bla*_*CTX-M-9-group*_ and *bla*_*CTX-M-1-group*_ genes were predominant in nosocomial (65.0%) and CA strains (66.7%), respectively. PFGE results showed ESBL-producing and CRKP strains were genetically diverse. Identical PFGE profiles were observed among HCA and nosocomial strains.

**Conclusions:**

Nosocomial and HCA *K. pneumoniae* infections presented similar clinical features and antimicrobial resistance, and both two types of infections were different to CA infections. CRKP and ESBL-producing strains were disseminated mainly in HCA and nosocomial groups, and showed a clonal diversity. The cross transmission of CRKP was existed among HCA and nosocomial patients. This finding suggests that similar empirical therapy should be considered for patients with nosocomial and HCA *K. pneumoniae* infections and bacterial resistance surveillance of these infections is necessary.

## Background

*Klebsiella pneumoniae* is a threatening human pathogen that causes various diseases, including respiratory tract and urinary tract infections, blood infections, and liver abscesses [1]. Traditionally, bacterial infections have been classified into community-acquired and nosocomial infections according to the route of infection. As the healthcare-associated effects on community-onset infections cannot be overlooked, these infections have been divided into community-onset healthcare-associated (HCA) infections for patients with a recent history of medical care, and community-acquired infections for patients without [2]. Previous studies have described that HCA infections are different from CA infections and share some similarities with nosocomial infections regarding clinical characteristics, outcome and microbiological features [2, 3]. Although there have been studies on HCA infections, the data about antimicrobial resistance and molecular features of HCA *K. pneumoniae* infections are limited [4, 5]. Inadequate understanding of these infections could lead to inappropriate empirical treatment and, in consequence, higher morbidity and mortality.

Antimicrobial resistance in *K. pneumoniae* is a significant concern to public health. The CRKP and ESBL-positive *K. pneumoniae* strains are the most important and challenging drug-resistant bacteria, leading to limited treatment options and high mortality [6–8]. Thus, understanding the distribution of such highly resistant strains in both hospitals and communities is vital to planning interventions.

The production of carbapenemase is the main carbapenem-resistance mechanism in *K. pneumoniae* [9]. Carbapenemase encoding genes reported worldwide mainly include *bla*_KPC_, *bla*_NDM_, and *bla*_OXA-48-like_ [10–12]. *Bla*_SHV_, *bla*_TEM_, *bla*_CTX-M_, and *bla*_OXA_ are the major ESBL genes identified to date [13, 14]. These antibiotic resistance genes are normally located on mobile genetic elements, such as plasmids and transposons, thus, they can be widely spread in both nosocomial and community environments [15, 16].

Strain typing is of great significance for epidemiological surveillance. PFGE is a gold standard for the genotyping of strains. The molecular typing of *K. pneumoniae* isolates contributes to the identification of specific clone dissemination and the source of infections, on which the prevention approach depends.

Currently, there are limited data comparing the clinical and microbiological characteristics of nosocomial, HCA, and CA *K. pneumoniae* infections. And the study data on antimicrobial resistance of HCA *K. pneumoniae* infections is scarce. Furthermore, strains isolated from different regions present their own distinct clinical and microbiological features. Therefore, this study aimed to characterize the clinical features, antimicrobial resistance and the distribution of CRKP and ESBL-positive strains among nosocomial, HCA, and CA infections caused by *K. pneumoniae,* and to characterize the genetic relatedness of CRKP and ESBL-positive isolates identified from the three different groups. The results from this study will be helpful in developing effective preventive and treatment strategies against *K. pneumoniae* infections both in hospitals and in communities.

## Methods

### Study design and population

This retrospective study was conducted at a 2300-bed tertiary hospital in Guangzhou, China. All patients presenting *K. pneumoniae* infections from January 2019 to December 2019 were included in the study. *K. pneumoniae* strains were consecutively collected from these patients. For patients with multiple *K. pneumoniae* isolates, only the first isolate was analysed. During the study period, the number of admittances and outpatient treatments were about 103,630 and 1,610,618, respectively.

The definitions of nosocomial, HCA, and CA *K. pneumoniae* infection was based on previously described criteria [2]. A nosocomial *K. pneumoniae* infection was defined as an infection that occurred 48 h after the patient’s admission. A HCA *K. pneumoniae* infection was defined as an infection developing within 48 h of admission in patients presenting any of the following risk factors [2]: a history of intravenous therapy or renal dialysis in the 30 days before the *K. pneumoniae* infection; a history of hospitalization for 2 or more days in the three months before the *K. pneumoniae* infection; or residence in a nursing home or long-term care facility. A CA *K. pneumoniae* infection was defined as an infection occurring in patients who did not fulfill the definitions of nosocomial or HCA infections.

### Clinical data collection

We extracted the following clinical data from electronic records for all patients: demographic characteristics, underlying disease, site of infection, immunosuppression, and mortality. Exposure to prior antimicrobial treatment was defined as any treatment received for at least 48 h in the 30 days before the *K. pneumoniae* infection.

### Bacterial identification and antimicrobial susceptibility testing

A total of 379 *K. pneumoniae* strains were collected from patients during the study period. The Vitek 2 system (bioMérieux, France) was used for bacterial identification. Antimicrobial susceptibility and the ESBL phenotype were determined using the Vitek 2 System following the Clinical and Laboratory Standards Institute guidelines (CLSI, 2019). The antibiotics tested included ampicillin (AMP), piperacillin/tazobactam (TZP), amoxicillin/clavulanic acid (AMC), cefoperazone/sulbactam (CPS), cefazolin (CZO), cefuroxime (CXM), cefepime (FEP), ceftriaxone (CRO), ciprofloxacin (CIP), cefoxitin (FOX), ceftazidime (CAZ), imipenem (IMP), amikacin (AMK), gentamicin (GEN), ertapenem (ERP), meropenem (MEM), aztreonam (ATM), levofloxacin (LVE), trimethoprim/sulfamethoxazole (SXT), and tigecycline (TGC). The breakpoint of tigecycline was based on the standard of the United States Food and Drug Administration (FDA). *Escherichia coli* ATCC 25,922 and *K. pneumoniae* ATCC 700,603 served as quality control strains.

Multidrug resistant (MDR) strains were defined as strains with no susceptibility to at least three different classes of antimicrobials [17]. Carbapenem-resistant *K. pneumoniae* (CRKP) isolates were defined as isolates that displayed resistance to one or more carbapenem agents such as meropenem, ertapenem, or imipenem [18].

### Detection of resistance genes

Polymerase chain reaction (PCR) was performed to detect carbapenemase genes (*bla*_KPC_*, bla*_NDM_*, bla*_GIM_*, bla*_SPM_*, bla*_IMP_*, bla*_SIM_*, bla*_VIM_*, bla*_IMI_ and *bla*_OXA-48-like_) in the CRKP strains and ESBLs genes (*bla*_TEM_*, bla*_SHV_*, bla*_CTX-M-group_*, bla*_OXA-1-like_, *bla*_CTX-M-1-group,_ *bla*_CTX-M-2-group_, *bla*_CTX-M-9-group_) in the ESBL-producing strains [17, 19–22]. The commercial kits Green Taq Mix (Vazyme, Nanjing, China) and specific primers were used to detect resistance genes. Amplification conditions are as follows: initial denaturation at 95℃ for 3 min, 30 cycles of 95℃ for 40 s, primer annealing temperature for 40 s, 72℃ for 50 s, and 72℃ for 7 min. Bacterial strains with resistance genes detected by PCR and DNA sequencing were used as positive controls for the subsequent PCR runs. The distilled water was used as a negative control. Amplification products were analyzed by agarose gel electrophoresis. The specific primers are presented in Additional file 1: Table S1. The PCR products were sequenced and the sequencing results were analyzed on the BLAST website (http://blast.ncbi.nlm.nih.gov).

### Pulsed-field gel electrophoresis (PFGE)

All 186 CRKP or ESBL-producing strains were subjected to PFGE following the PulseNet standardized procedure (http://www.cdc.gov/pulsenet/protocols.htm). Briefly, the strain DNA were digested with *Xbal* for 2 h at 37 ℃. Electrophoresis was performed using the CHEF-DRIII system (120 angle, 6 V/cm), with a running time of 19 h and a switch time of 2.16–54 s. The PFGE fingerprint patterns were uploaded to the *K. pneumoniae* database of the Chinese Pathogen Identification Net (http://139.9.117.189/CPIN/). A Dice coefficient-based PFGE dendrogram was constructed using the unweighted pair-group mean Analysis. Isolates were grouped into the same PFGE cluster if they shared ≥ 80% similarity.

### Statistical analyses

Categorical variables were analyzed by Chi-square or Fisher’s exact tests (SPSS software, version 20.0). *P-*values < 0.05 (two-tailed) were considered statistically significant. Multivariable logistic regression analysis was conducted to identify variables associated with nosocomial, HCA, and CA infections. Variables regarding age, underlying disease, and immunosuppression with *P* < 0.05 in the univariate analysis were included in the multivariable logistic regression model.

## Results

### Clinical features of *K. pneumoniae* infection

In total, 379 patients with *K. pneumoniae* infection were included (98 nosocomial, 195 HCA and 86 CA infections) in the analysis. The incidence of *K. pneumoniae* infections was approximately 3.66 per 1000 admissions in the hospital. Male patients accounted for 64.12% of *K. pneumoniae* infections. The overall 30-day crude mortality rate was 12.4% (47/379).

The clinical features of *K. pneumoniae* infections are presented in Table 1. When compared to HCA (6.2%) and CA infections (5.8%), nosocomial infections (21.4%) occurred more frequently in patients aged ≤ 1 years old. Multivariate analysis further showed that patients aged ≤ 1 years old (nosocomial vs. HCA: OR = 5.665, nosocomial vs. CA: OR = 4.456) was an independent variable associated with nosocomial infections (Table 2).

**Table 1:** Clinical features of *K. pneumoniae* infections

Variable	Nosocomial	HCA	CA	*P*-value		
	(n = 98)	(n = 195)	(n = 86)	HCA versus Nosocomial	CA versus Nosocomial	CA versus HCA
Age (years)						
≤ 1	21 (21.4%)	12 (6.2%)	5 (5.8%)	**0.000**	**0.002**	0.912
2–10	10 (10.2%)	5 (2.6%)	1 (1.2%)	**0.005**	**0.010**	0.671
11–18	8 (8.2%)	1 (0.5%)	2 (2.3%)	**0.001**	0.107	0.223
19–59	34 (34.7%)	89 (45.6%)	48 (55.8%)	0.073	**0.004**	0.116
≥ 60	25 (25.5%)	88 (45.1%)	30 (34.9%)	**0.001**	0.166	0.109
Sex						
Male	56 (57.1%)	131 (67.2%)	56 (65.1%)	0.092	0.269	0.736
Infection type						
Respiratory tract	23 (23.5%)	66 (33.9%)	18 (20.9%)	0.068	0.68	**0.029**
Bloodstream	45 (45.9%)	35 (18.0%)	7 (8.1%)	**0.000**	**0.000**	**0.034**
Urinary tract	9 (9.2%)	46 (23.6%)	18 (20.9%)	**0.003**	**0.025**	0.624
Cerebra	5 (5.1%)	3 (1.5%)	0 (0.0%)	0.123	**0.034**	0.555
Skin and soft tissue	3 (3.1%)	16 (8.2%)	22 (25.6%)	0.092	**0.000**	**0.000**
Oral cavity	5 (5.1%)	3 (1.5%)	1 (1.2%)	0.123	0.217	1.000
Other	8 (8.2%)	16 (8.2%)	11 (12.8%)	0.990	0.303	0.229
liver	0 (0.0%)	1 (0.5%)	4 (4.7%)	1.000	**0.031**	**0.032**
Biliary tract	0 (0.0%)	9 (4.6%)	5 (5.8%)	**0.032**	**0.016**	0.767
Underlying disease						
Solid tumor	7 (7.1%)	20 (10.3%)	5 (5.8%)	0.385	0.716	0.264
Diabetes mellitus	8 (8.2%)	34 (17.4%)	24 (27.9%)	**0.033**	**0.000**	0.055
Pulmonary disease	1 (1.0%)	7 (3.6%)	5 (5.8%)	0.276	0.099	0.522
Hematological malignancy	25 (25.5%)	23 (11.8%)	3 (3.5%)	**0.003**	**0.000**	**0.027**
Brain tumor	10 (10.2%)	7 (3.6%)	2 (2.3%)	**0.022**	**0.031**	0.727
Cerebral vascular disease	13 (13.3%)	47 (24.1%)	10 (11.6%)	**0.030**	0.738	**0.017**
Hepatobiliary disease	7 (7.1%)	13 (6.7%)	8 (9.3%)	0.879	0.593	0.439
Chronic kidney disease	4 (4.1%)	21 (10.8%)	7 (8.1%)	0.053	0.247	0.498
Cardiovascular disease	4 (4.1%)	20 (10.3%)	5 (5.8%)	0.069	0.736	0.228
Hypertension	18 (18.4%)	61 (31.3%)	17 (19.8%)	**0.019**	0.852	**0.047**
Immunosuppression	29 (29.6%)	38 (19.5%)	6 (7.0%)	0.052	**0.000**	**0.008**
Prior antibiotic exposure						
Any antibiotic	53 (54.1%)	115 (59.0%)	0 (0.0%)	0.424	**0.000**	**0.000**
1st or 2nd generation cephalosporin	6 (6.1%)	7 (3.6%)	0 (0.0%)	–	–	–
3rd or 4th Generation cephalosporin	10 (10.2%)	17 (8.7%)	0 (0.0%)	–	–	–
β-lactam and β-lactamase inhibitor	14 (14.3%)	19 (9.7%)	0 (0.0%)	–	–	–
Carbapenem	17 (17.4%)	21 (10.8%)	0 (0.0%)	–	–	–
Fluoroquinolone	2 (2.0%)	5 (2.6%)	0 (0.0%)	–	–	–
Aminoglycoside	2 (2.0%)	3 (1.5%)	0 (0.0%)	–	–	–
Tigecycline	3 (3.1%)	3 (1.5%)	0 (0.0%)	–	–	–
Glycopeptide	7 (7.1%)	12 (6.2%)	0 (0.0%)	–	–	–
Metronidazole	0 (0.0%)	1 (0.5%)	0 (0.0%)	–	–	–
30-day crude mortality	19 (19.4%)	24 (12.3%)	4 (4.7%)	0.106	**0.003**	**0.048**

Data are presented as number (%)

Other included renal drainage fluid, amniotic fluid, ascites, and pleural fluid specimen

Bold values suggest statistical significance

^−^ not applicable

**Table 2:** Multivariate analysis of variables associated with *K. pneumoniae* infections

variable	Univariate OR (95% CI)	*P*-value	Mutivariate OR (95% CI)	*P*-value
Nosocomial versus HCA				
≤ 1	6.160 (2.668–14.224)	0.000	**5.665 (2.277–14.092)**	**0.000**
2–10	7.040 (2.203–22.497)	0.001	**5.332 (1.506–18.870)**	**0.009**
11–18	24.640 (2.894–209.815)	0.003	**18.772 (2.09–168.583)**	**0.009**
19–59	1.384 (0.766–2.502)	0.282	1.308 (0.699–2.445)	0.401
Diabetes mellitus	0.037 (0.187–0.948)	0.037	0.609 (0.249–1.487)	0.276
Hematological malignancy	2.425 (1.287–4.570)	0.006	1.882 (0.686–5.166)	0.220
Brain tumor	3.052 (1.124–8.284)	0.029	**3.411 (1.139–10.215)**	**0.028**
Cerebral vascular disease	0.525 (0.273–1.009)	0.053	0.841 (0.395–1.792)	0.654
Hypertension	0.528 (0.294–0.949)	0.033	1.189 (0.592–2.391)	0.626
Immunosuppression	1.653 (0.941–2.904)	0.081	0.912 (0.37–2.249)	0.912
Nosocomial versus CA				
≤ 1	5.040 (1.660–15.299)	0.004	**4.456 (1.405–14.135)**	**0.011**
2–10	12.000 (1.436–100.278)	0.022	4.727 (0.494–45.221)	0.178
11–18	4.800 (0.933–24.692)	0.061	1.981 (0.327–12.001)	0.457
19–59	0.850 (0.427–1.693)	0.644	0.671 (0.312–1.442)	0.307
Diabetes mellitus	0.230 (0.097–0.544)	0.001	**0.342 (0.129–0.906)**	**0.031**
Hematological malignancy	9.475 (2.747–32.681)	0.000	**4.467 (0.933–21.395)**	**0.061**
Brain tumor	4.773 (1.016–22.427)	0.048	**7.865 (1.554–39.817)**	**0.013**
Immunosuppression	5.604 (2.197–14.291)	0.000	2.762 (0.807–9.461)	0.106
HCA versus CA				
Diabetes mellitus	0.546 (0.300–0.993)	0.047	0.554 (0.288–1.067)	0.077
Hematological malignancy	3.700 (1.080–12.674)	0.037	2.62 (0.664–10.344)	0.169
Cerebral vascular disease	2.414 (1.156–5.041)	0.019	**2.486 (1.154–5.356)**	**0.02**
Hypertension	1.848 (1.003–3.404)	0.049	**2.08 (1.065–4.061)**	**0.032**
Immunosuppression	3.227 (1.309–7.954)	0.011	2.661 (0.971–7.297)	0.057

Bold values suggest statistical significance

There were differences between the three groups regarding the infection type, with bloodstream infections being dominant in nosocomial patients (45.9%), respiratory tract infections being frequent in HCA patients (33.9%), and skin and soft tissue infections being predominant in CA patients (25.6%).

Nosocomial infections and HCA infections shared similar features in terms of underlying diseases. However, CA infections differed from both nosocomial and HCA infections. In multivariate analysis, hematological malignancy (OR = 4.467) and brain tumor (OR = 7.865) were independent variables associated with nosocomial cases compared to CA cases, whereas diabetes mellitus was more frequently associated with CA cases (OR = 0.342). Cerebral vascular disease (OR = 2.486) and hypertension (OR = 2.080) were independent variables associated with HCA infections compared to CA infections.

Both patients with nosocomial and HCA infections presented a higher prevalence of prior antibiotic exposure, accounting for 54.1% and 59.0%, respectively. No prior antibiotic exposure was observed in CA infection patients. Patients with nosocomial (19.4%) and HCA (12.3%) infections exhibited higher rates of 30-day mortality than patients with CA infections (4.7%).

### Antimicrobial resistance of nosocomial, HCA, and CA *K. pneumoniae* isolates

The distribution of the percentage of antimicrobial-resistant *K. pneumoniae* strains isolated from the three groups is summarized in Table 3. Overall, the resistance rates to most of the antibiotics tested were similar for nosocomial isolates and HCA isolates (*P* > 0.05), and both the strains were significantly more resistant than the CA strains (*P* < 0.05). The percentages of CRKP strains, ESBL-producing strains and MDR strains were similar between the nosocomial and HCA cases and were higher in both cases than in CA cases: (nosocomial vs. HCA vs. CA) for CRKP strains (22.5% vs. 28.7% vs. 4.7%), ESBL-producing strains (40.8% vs. 35.9% vs. 24.4%), and MDR strains (53.1% vs. 52.3% vs. 23.3%).

**Table 3:** Antimicrobial resistance rates of *K. pneumoniae* strains

Antimicrobials	Nosocomial	HCA	CA	*P*-value		
	(n = 98)	(n = 195)	(n = 86)	HCA versus Nosocomial	CA versus Nosocomial	CA versus HCA
Ampicillin	96 (98.0%)	195 (100.0%)	82 (95.4%)	0.111	0.420	0.002
Cefazolin	68 (69.4%)	131 (67.2%)	36 (41.9%)	0.702	**0.000**	**0.000**
Cefuroxime	58 (59.2%)	114 (58.5%)	27 (31.4%)	0.906	**0.000**	**0.000**
Ceftriaxone	56 (57.1%)	109 (55.9%)	26 (30.2%)	0.839	**0.000**	**0.000**
Ceftazidime	43 (43.9%)	85 (43.6%)	18 (20.9%)	0.963	**0.001**	**0.000**
Cefepime	41 (41.8%)	87 (44.6%)	15 (17.4%)	0.651	**0.000**	**0.000**
Imipenem	20 (20.4%)	57 (29.2%)	4 (4.7%)	0.105	**0.002**	**0.000**
Meropenem	21 (21.4%)	57 (29.2%)	4 (4.7%)	0.154	**0.001**	**0.000**
Ertapenem	24 (24.5%)	58 (29.7%)	4 (4.7%)	0.345	**0.000**	**0.000**
Amoxicillin/Clavulanic Acid	44 (44.9%)	78 (40.0%)	13 (15.1%)	0.422	**0.000**	**0.000**
Cefoperazone/Sulbactam	41 (41.8%)	66 (33.9%)	9 (10.5%)	0.180	**0.000**	**0.000**
Piperacillin/ Tazobactam	43 (43.9%)	74 (38.0%)	8 (9.3%)	0.328	**0.000**	**0.000**
Aztreonam	47 (48.0%)	102 (52.3%)	22 (25.6%)	0.482	**0.002**	**0.000**
Cefoxitin	31 (31.6%)	73 (37.4%)	11 (12.8%)	0.327	**0.002**	**0.000**
Ciprofloxacin	45 (45.9%)	103 (52.8%)	21 (24.4%)	0.265	**0.002**	**0.000**
Levofloxacin	31 (31.6%)	90 (46.2%)	14 (16.3%)	**0.017**	**0.016**	**0.000**
Amikacin	36 (36.7%)	46 (23.6%)	6 (7.0%)	**0.018**	**0.000**	**0.001**
Gentamicin	47 (48.0%)	91 (46.6%)	24 (27.9%)	0.834	**0.005**	**0.004**
Trimethoprim/Sulfamethoxazole	52 (53.1%)	106 (54.4%)	34 (39.5%)	0.833	0.067	**0.028**
Tigecycline	1 (1.0%)	4 (2.1%)	1 (1.2%)	0.667	1.000	1.000
ESBL-production	40 (40.8%)	70 (35.9%)	21 (24.4%)	0.412	**0.018**	0.072
Multidrug resistance	52 (53.1%)	102 (52.3%)	20 (23.3%)	0.903	**0.000**	**0.000**

Data are presented as number (%)

Bold values suggest statistical significance

### Distribution of carbapenem-resistance genes and ESBL genes

A total of 82 CRKP isolates were identified from the three groups. The distribution of carbapenem resistance genes of the CRKP isolates among the three groups is shown in Table 4. Only 3 carbapenem resistance genes, *bla*_KPC_ (80.5%, 66/82), *bla*_NDM_ (14.6%, 12/82), and *bla*_OXA-48-like_ (3.7%, 3/82), were identified among the 82 CRKP strains. Four strains presented simultaneously *bla*_KPC_ and *bla*_NDM_ genes. Five isolates were negative for the screened carbapenemase genes.

**Table 4:** Carbapenemase resistance genes distribution among CRKP strains

Carbapenemase genes	Total	Nosocomial	HCA	CA	*P*-value		
	(n = 82)	(n = 22)	(n = 56)	(n = 4)	HCA versus Nosocomial	CA versus Nosocomial	CA versus HCA
*bla*_KPC_	66 (80.5%)	19 (86.4%)	44 (78.6%)	3 (75.0%)	0.563	0.511	1.000
*bla*_NDM_	12 (14.6%)	2 (9.1%)	9 (16.1%)	1 (25.0%)	0.719	0.408	0.528
*bla*_OXA-48-like_	3 (3.7%)	1 (4.6%)	2 (3.6%)	0 (0.0%)	–	–	–
*bla*_KPC_ and *bla*_NDM_	4 (4.9%)	1 (4.6%)	3 (5.4%)	0 (0.0%)	–	–	–
None detected	5 (6.1%)	1 (4.6%)	4 (7.1%)	0 (0.0%)	–	–	–

Data are presented as number (%)

^−^: not applicable

PCR showed that all nosocomial, HCA, and CA ESBL-positive strains harbored at least one of the ESBL gene groups, with positive rates of 91.6% (120/131) for *bla*_SHV_, 64.9% (85/131) for *bla*_TEM_, 51.9% (68/131) for *bla*_CTX-M-9-group_, 42.0% (55/131) for *bla*_CTX-M-1-group_ (Table 5). The *bla*_CTX-M-2-group_ gene was not detected in ESBL-producing strains. No significant differences were found in the distribution of *bla*_SHV_, *bla*_TEM_, and *bla*_OXA-1-like_ genes among the nosocomial, HCA, and CA infection groups. However, the *bla*_CTX-M-1-group_ and *bla*_CTX-M-9-group_ genes were disproportionately distributed between nosocomial, HCA, and CA isolates (Table 5). Fifty-one of the 131 ESBL-positive strains were observed to co-harbor *bla*_SHV_ and *bla*_CTX-M-1-group_, with 74.5% (38/51) having co-existence of *bla*_SHV_, *bla*_TEM_ and *bla*_CTX-M-1-group,_ and these strains were more commonly identified in the CA group than in the nosocomial group (61.9% vs. 20.0%,* P* = 0.001; 42.9% vs. 17.5%, *P* = 0.032, respectively). Conversely, strains possessing both *bla*_SHV_ and *bla*_CTX-M-9-group_ were more likely to be found in the nosocomial infection group than in the CA group (55.0% vs. 23.8%, *P* = 0.02).

**Table 5:** Distribution of ESBLs genes among three groups of ESBL-producing strains

ESBLs genes	Total	Nosocomial	HCA	CA	*P*-value		
	(n = 131)	(n = 40)	(n = 70)	(n = 21)	HCA versus	CA versus	CA versus
					Nosocomial	Nosocomial	HCA
*bla*_SHV_	120 (91.6%)	34 (85.0%)	67 (95.7%)	19 (90.5%)	0.070	0.703	0.326
*bla*_TEM_	85 (64.9%)	25 (62.5%)	45 (64.3%)	15 (71.4%)	0.851	0.486	0.545
*bla*_OXA-1-like_	6 (4.6%)	0 (0.0%)	5 (7.1%)	1 (4.8%)	0.157	0.344	1.000
*bla*_CTX-M-group_	123 (93.9%)	39 (97.5%)	65 (92.9%)	19 (90.5%)	0.414	0.27	0.66
*bla*_CTX-M-1-group_	55 (42.0%)	10 (25.0%)	31 (44.3%)	14 (66.7%)	**0.044**	**0.002**	0.072
*bla*_CTX-M-9-group_	68 (51.9%)	26 (65.0%)	36 (51.4%)	6 (28.6%)	0.167	**0.007**	0.065
*bla*_SHV_ and *bla*_CTX-M-1-group_	51 (38.9%)	8 (20.0%)	30 (42.9%)	13 (61.9%)	**0.015**	**0.001**	0.125
*bla*_SHV_ and *bla*_CTX-M-9-group_	61 (46.6%)	22 (55.0%)	34 (48.6%)	5 (23.8%)	0.516	**0.020**	**0.044**
*bla*_TEM_ and *bla*_CTX-M-1-group_	42 (32.1%)	9 (22.5%)	23 (32.9%)	10 (47.6%)	0.250	0.440	0.217
*bla*_TEM_ and *bla*_CTX-M-9-group_	40 (30.5%)	15 (37.5%)	21 (30.0%)	4 (19.1%)	0.420	0.139	0.324
*bla*_CTX-M-9-group_ and *bla*_CTX-M-1-group_	8 (6.1%)	2 (5.0%)	5 (7.1%)	1 (4.8%)	–	–	–
*bla*_SHV_, *bla*_TEM_ and *bla*_CTX-M-9-group_	36 (27.5%)	13 (32.5%)	20 (28.6%)	3 (14.3%)	0.665	0.124	0.186
*bla*_SHV_, *bla*_TEM_ and *bla*_CTX-M-1-group_	38 (29.0%)	7 (17.5%)	22 (31.4%)	9 (42.9%)	0.111	**0.032**	0.332
*bla*_SHV_, *bla*_CTX-M-9-group_ and *bla*_CTX-M-1-group_	8 (6.1%)	2 (5.0%)	5 (7.1%)	1 (4.8%)	–	–	–
*bla*_TEM_, *bla*_CTX-M-9-group_ and *bla*_CTX-M-1-group_	6 (4.6%)	2 (5.0%)	3 (4.3%)	1 (4.8%)	–	–	–
*bla*_SHV_, *bla*_TEM_, *bla*_CTX-M-9-group_ and *bla*_CTX-M-1-group_	6 (4.6%)	2 (5.0%)	3 (4.3%)	1 (4.8%)	–	–	–

Data are presented as number (%)

Bold values suggest statistical significance

^−^ not applicable

### Pulsed-field gel electrophoresis

**Fig. 1:**
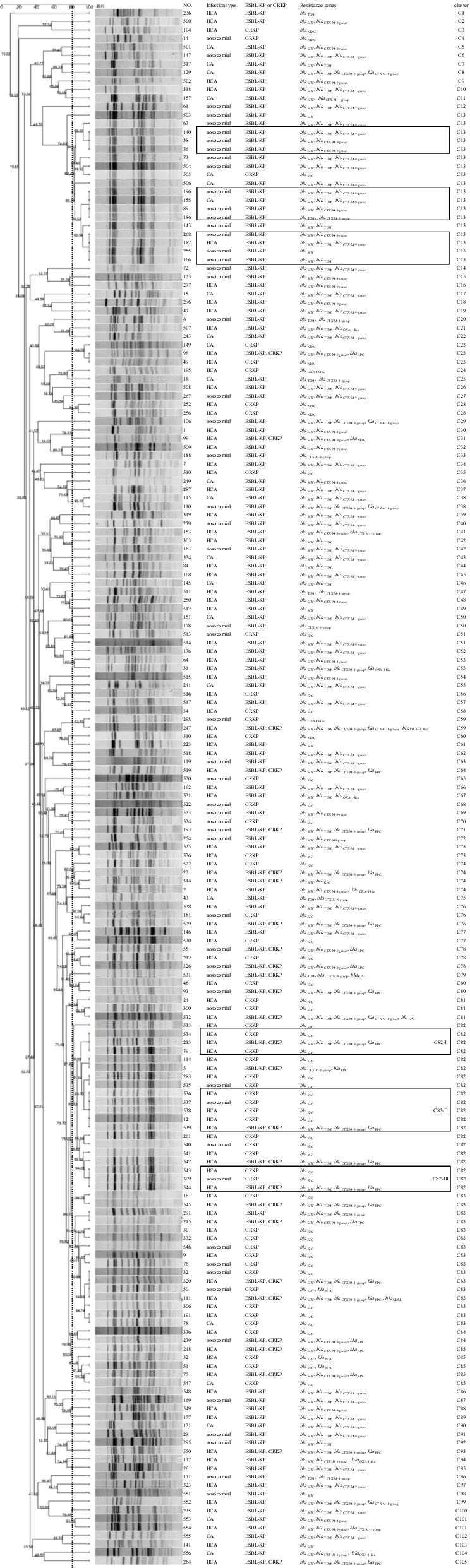
PFGE analysis of ESBL-producing and carbapenem-resistant *K. pneumoniae* strains. HCA: Healthcare-associated; CA: Community-acquired; CRKP: carbapenem-resistant *K. pneumoniae*; ESBL-KP: ESBL-producing *K. pneumoniae*

All CRKP or ESBL-producing strains were subjected to PFGE for assessing the clonality of the strains. Three ESBL-producing isolates failed to be genotyped by PFGE. Fifty-six nosocomial strains were divided into 36 clusters at a cut-off of 80% similarity, with the major cluster C13 accounting for 14 ESBL-producing strains (Fig. 1). One hundred and three HCA strains were separated into 65 clusters, with two major clusters C82 and C83, accounting for 17 and 10 CRKP strains respectively. Twenty-four CA strains distributed among 22 clusters. Despite the high genetic diversity, several sets of identical profiles were found among the three groups. In PFGE cluster C13, three sets of identical profiles were observed, and 9 of which were nosocomial strains. In PFGE cluster C82, two sets of identical PFGE profiles were detected among HCA isolates. In cluster C82-II, four HCA isolates and one nosocomial strain showed 100% similarity. Two HCA isolates and one nosocomial strain in cluster C82-III also shared complete similarity. In addition, we observed that some strains with the same PFGE profiles but carried different resistance gene profiles. For instance, isolate 268, 182, 255, and 166 in cluster C13 presented identical profiles, but carried different *bla* genes. Isolate 98 carrying *bla*_KPC_ showed 100% similarity to isolate 49 carrying *bla*_NDM_.

## Discussion

In the current study, we used the definition proposed by Friedman et al. [2] to define healthcare-associated *K. pneumoniae* infections, and we found that HCA infections accounted for over half of the *K. pneumoniae* infections. HCA infections showed similar clinical features with nosocomial infections. CA infections presented different characteristic from both HCA and nosocomial infections. Hematological malignancy was more commonly related to nosocomial infections, whereas diabetes mellitus was more frequently associated with CA infections. These findings were also observed in previous reports from Taiwan and Korea [23, 24]. It has been reported that cancer was more commonly associated with HCA *K. pneumoniae* bacteremia patients than CA bacteremia patients [4, 5, 25]. In the present study, however, cerebral vascular disease and hypertension were more associated with HCA infections compared to CA infections. This discrepancy may be due to the regional differences in underlying diseases. Indeed, cerebral vascular disease and hypertension are epidemics in Chinese communities [26, 27]. Patients with these diseases have a greater demand for medical care services.

In this study, the 30-day mortality rates were similar for patients with nosocomial and HCA infections and both patients exhibited significantly higher 30-day mortality rates than CA infection patients. These results were similar to other studies [4, 5] and may largely be due to the variation in underlying diseases between the three groups, which are classical risk factors for prognosis of infectious diseases.

An important finding in this study was that HCA *K. pneumoniae* strains exhibited similar antimicrobial resistance patterns with nosocomial strains, and both the nosocomial and HCA strains were significantly more resistant than the CA strains. This observation indicates that clinicians should consider similar empirical therapy for patients with nosocomial and HCA *K. pneumoniae* infections. A recent study in Taiwan reported that the rate of resistance in the group of HCA *K. pneumoniae* bacteremia was between those groups of nosocomial and CA bacteremia [28]. This discrepancy may be due to the different infection types of the population studied, or the regional differences of resistance characteristics in *K. pneumoniae*. In our study, 29.7% of the HCA isolates were carbapenem resistant, 35.9% expressed ESBL, and 52.3% were MDR, indicating the severe drug resistance in isolates from the HCA infections. The high degree of antibiotic resistance in HCA strains may be related to the frequent contact with healthcare facilities among HCA patients, where the drug-resistant bacteria could spread. In addition, other factors such as overuse of antibiotics might also responsible for developing antimicrobial resistance. Our results suggest that increased attention should be paid to the HCA *K. pneumoniae* infections.

The spread of CRKP strains has become a new public health crisis worldwide. Published studies on the distribution of CRKP isolates among nosocomial, HCA, and CA infections are scarce. The present study showed that CRKP strains were rare in the CA group, but more prevalent in both HCA and nosocomial groups. Thus, it should be of caution when use carbapenems to treat patients with HCA and nosocomial infections. In agreement with other studies, the *bla*_KPC_ was the dominant carbapenemase gene carried by CRKP strains [18, 29]. In this investigation, only 12 and 3 CRKP strains harbored *bla*_NDM_ and *bla*_OXA-48-like_ genes respectively. Indeed, the *bla*_OXA-48-like_ producers were more prevalent in western countries such as France, Spain and Germany, but were rare in China [30–32]. In addition, the *bla* genes screened were absent in five CRKP isolates. Among these strains, only one showed carbapemase activity in the modified carbapenem inactivation method test (data not shown), indicating other resistance genes not investigated may mediate the carbapemase activity. The remaining four CRKP may have other resistance mechanisms contributing to carbapenem resistance. The production of ESBLs combined with mutations in or loss of the porin Ompk35 or Ompk36 can be associated with carbapenem resistance [33, 34]. In addition, the overexpression of efflux pump in strains may also lead to carbapenem resistance. However, both porin Ompk35 and Ompk36 were present in the other four CRKP strains with ESBL-positive in this study (data not shown). Further studies should be conducted to assess the presence of the mutations in strains isolated in the current investigation.

This study showed that the ESBL-producing strains were prevalent both in medical and community settings. Previous studies by Zhang et al. [35] and Quan et al. [36] also reported a high prevalence of ESBL-positive strains in community-onset *K. pneumoniae* infections. These data suggest that ESBL producers have disseminated to the community, which poses a challenge to resistance control. *Bla*_CTX-M_ was the most frequently identified ESBL gene among ESBL producers in our study. This is accordance with the fact that CTX-M is the most common ESBL genotype in China [36]. No significant differences were observed in the distribution of *bla*_CTX-M_, *bla*_SHV_ and *bla*_TEM_ genes among isolates from the three groups, suggesting the wide dissemination of these genes possibly via horizontal transfer in different populations. Interestingly, the CTX-M-1 variant was more frequently detected among CA strains than among nosocomial strains, whereas the CTX-M-9 variant was more common in nosocomial strains than in CA strains. This suggests that nosocomial and CA ESBL-producing strains may carry different plasmids, which carried the different subtype of CTX-M genes.

Overall, the PFGE typing showed a high clonal diversity among the ESBL-producing and CRKP strains isolated from the three different groups, indicating the variety of infection sources acquired by the three populations. Of note, the clonal dissemination of ESBL-producing strains has occurred in the hospital as indicated by the three identical profiles observed in cluster C13. Further study is needed to investigate the origin and the route of the transmission. In cluster C82-II and cluster C82-III, the HCA strains and nosocomial strains showed the same PFGE profiles, suggesting the cross transmission of CRKP among HCA and nosocomial patients. CRKP strains could be introduced into the hospital, then spread within the institution and might cause nosocomial infections during the entry of HCA patients with CRKP infections, of which we should keep alert. Identical profiles among HCA CRKP isolates implies the clonal spread of *K. pneumoniae* among HCA patients. It suggests the healthcare-associated transmission of CRKP strains in the local area. In addition, the same PFGE profiles were observed in strains carrying different resistance gene profiles, suggesting later acquisition of the resistance genes via horizontal transfer under antibiotic selection pressure.

The main finding in the present study was that the antimicrobial resistance of HCA isolates was similar in severity to nosocomial isolates. This study was conducted at a tertiary hospital in Guangzhou city, Guangdong province, where more severe patients from all over the province or neighboring provinces had been hospitalized and approximately 20% had been referred from other hospitals, which could explain the serious drug resistance in the HCA group in our study. Given distinct features of *K. pneumoniae* strains from different regions, more epidemiological data on nosocomial, HCA, and CA *K. pneumoniae* infections are needed to better manage patients.

## Conclusions

HCA *K. pneumoniae* infections were similar to nosocomial infections regarding clinical features and antimicrobial resistance, and were different to CA infections. CRKP and ESBL-producing strains were mainly prevalent in patients with HCA and nosocomial infections, showing genetic diversity. Identical profiles observed in HCA and nosocomial CRKP strains suggested the transmission of CRKP among HCA and nosocomial patients. These findings indicate that empirical antimicrobial treatment for patients with HCA *K. pneumoniae* infections should be similar to those for patients with nosocomial infections and great attention should be paid to these infections due to the possible dissemination of antimicrobial resistance among them.

## Supplementary Information

**Additional file 1: Table S1.** Primer sequences of resistant genes for *K. pneumoniae*.

## Data Availability

All materials and data analyzed during this study are contained within the manuscript.

## Abbreviations

PCR: Polymerase chain reaction
CRKP: Carbapenem-resistant *K. pneumoniae*
ESBL: Extended-spectrum beta-lactamase
MDR: Multidrug resistant
PFGE: Pulsed-field gel electrophoresis
HCA: Healthcare-associated
CA: Community-acquired
