# Supplementary material for: Clinical and microbiological characteristics of nosocomial, healthcare-associated, and community-acquired Klebsiella pneumoniae infections in Guangzhou, China
Source: Antimicrob Resist Infect Control. 2021 Feb 25;10:41. doi: 10.1186/s13756-021-00910-1 (PMC7908793; doi:10.1186/s13756-021-00910-1)
Supplement: Supplementary file 1 — Additional file 1: Table S1. Primer sequences of resistant genes for K. pneumoniae. [file 13756_2021_910_MOESM1_ESM.doc]

**Supplemental Table 1 : Primer sequences of resistant genes for *K. pneumoniae***

| **Genes** | **Primers** | **Sequences (5’-3’)** | **Amplicon size(bp)** | **Fragments (bp)** | **References** |
| --- | --- | --- | --- | --- | --- |
| *bla*_kpc_ | KPC-F | CATTCAAGGGCTTTCTTGCTGC | 55℃ | 538 | [1] |
|  | KPC-R | ACGACGGCATAGTCATTTGC |  |  |  |
| *bla*_NDM_ | NDM-F | GCAGCTTGTCGGCCATGCGGGC | 60℃ | 782 | [2] |
|  | NDM-R | GGTCGCGAAGCTGAGCACCGCAT |  |  |  |
| *bla*_OXA-48-like_ | OXA-48-like-F | GCAAAACGCCGGGTTATTC | 55℃ | 438 | [2] |
|  | OXA-48-like-R | GGTTAGCGTTGCCAGTGCT |  |  |  |
| *bla*_IMP_ | IMP-F | CTACCGCAGCAGAGTCTTTGC | 55℃ | 587 | [2] |
|  | IMP-R | ACAACCAGTTTTGCCTTACC |  |  |  |
| *bla*_VIM_ | VIM-F | AAAGTTATGCCGCACTCACC | 55℃ | 865 | [2] |
|  | VIM-R | TGCAACTTCATGTTATGCCG |  |  |  |
| *bla*_GIM_ | GIM-F | TCGACACACCTTGGTCTGAA | 58.5℃ | 477 | [3] |
|  | GIM-R | AACTTCCAACTTTGCCATGC |  |  |  |
| *bla*_SPM_ | SPM-F | AAAATCTGGGTACGCAAACG | 59℃ | 271 | [3] |
|  | SPM-R | ACATTATCCGCTGGAACAGG |  |  |  |
| *bla*_SIM_ | SIM-F | TACAAGGGATTCGGCATCG | 61℃ | 570 | [3] |
|  | SIM-R | TAATGGCCTGTTCCCATGTG |  |  |  |
| *bla*_IMI_ | IMI-F | TGCGGTCGATTGGAGATAAA | 50℃ | 399 | [4] |
|  | IMI-R | CGATTCTTGAAGCTTCTGCG |  |  |  |
| *bla*_TEM_ | TEM-F | CATTTCCGTGTCGCCCTTATTC | 56℃ | 800 | [2] |
|  | TEM-R | CGTTCATCCATAGTTGCCTGAC |  |  |  |
| *bla*_SHV_ | SHV-F | AGCCGCTTGAGCAAATTAAAC | 56℃ | 713 | [2] |
|  | SHV-R | ATCCCGCAGATAAATCACCAC |  |  |  |
| *bla*_CTX-M_ | CTX-M-F | ATGTGCAGYACCAGTAARGTKATGGC | 58℃ | 593 | [3] |
|  | CTX-M-R | TGGGTRAARTARGTSACCAGAAYCAGCGG |  |  |  |
| *bla*_CTX-M-1-group_ | CTX-M-1-group-F | TTAGGAARTGTGCCGCTGYA | 56℃ | 688 | [2] |
|  | CTX-M-1-group-R | CGATATCGTTGGTGGTRCCAT |  |  |  |
| *bla*_CTX-M-2-group_ | CTX-M-2-group-F | CGTTAACGGCACGATGAC | 56℃ | 404 | [2] |
|  | CTX-M-2-group-R | CGATATCGTTGGTGGTRCCA |  |  |  |
| *bla*_CTX-M-9-group_ | CTX-M-9-group-F | TCAAGCCTGCCGATCTGGT | 56℃ | 561 | [2] |
|  | CTX-M-9-group-R | TGATTCTCGCCGCTGAAG |  |  |  |
| *bla*_OXA-1-like_ | OXA-1-like-F | GGCACCAGATTCAACTTTCAAG | 56℃ | 564 | [2] |
|  | OXA-1-like-R | GACCCCAAGTTTCCTGTAAGTG |  |  |  |

1. Dallenne C, Da Costa A, Decré D, Favier C, Arlet G. Development of a set of multiplex PCR assays for the detection of genes encoding important beta-lactamases in *Enterobacteriaceae*. J Antimicrob Chemother. 2010;65:490-495.

2. Ferreira RL, da Silva BCM, Rezende GS, Nakamura-Silva R, Pitondo-Silva A, Campanini EB, et al. High Prevalence of Multidrug-Resistant *Klebsiella pneumoniae* Harboring Several Virulence and β-Lactamase Encoding Genes in a Brazilian Intensive Care Unit. Front Microbiol. 2018;9:3198.

3. Kiaei S, Moradi M, Hosseini-Nave H, Ziasistani M, Kalantar-Neyestanaki D. Endemic dissemination of different sequence types of carbapenem-resistant *Klebsiella pneumoniae* strains harboring *bla_NDM_* and *16S rRNA* methylase genes in Kerman hospitals, Iran, from 2015 to 2017. Infect Drug Resist. 2019;12:45-54.

4. Hong SS, Kim K, Huh JY, Jung B, Kang MS, Hong SG: Multiplex PCR for rapid detection of genes encoding class A carbapenemases. Annals of laboratory medicine 2012, 32(5):359-361.
